# Family history of cancer and childhood rhabdomyosarcoma: a report from the Children's Oncology Group and the Utah Population Database

**DOI:** 10.1002/cam4.448

**Published:** 2015-03-23

**Authors:** Philip J Lupo, Heather E Danysh, Sharon E Plon, Karen Curtin, David Malkin, Simone Hettmer, Douglas S Hawkins, Stephen X Skapek, Logan G Spector, Karin Papworth, Beatrice Melin, Erik B Erhardt, Seymour Grufferman, Joshua D Schiffman

**Affiliations:** 1 Section of Hematology-Oncology, Department of Pediatrics, Baylor College of MedicineHouston, Texas, USA; 2 Center for Children's Cancer Research (C3R), University of Utah Health Sciences CenterSalt Lake City, Utah, USA; 3 Department of Internal Medicine, University of Utah School of MedicineSalt Lake City, Utah, USA; 4 Division of Hematology/Oncology, Department of Pediatrics, The Hospital for Sick Children, University of TorontoToronto, Canada; 5 Charité, University Hospital BerlinGermany; 6 Seattle Children's Hospital, University of WashingtonSeattle, Washington, USA; 7 Fred Hutchinson Cancer Research CenterSeattle, Washington, USA; 8 Division of Hematology/Oncology, Department of Pediatrics, University of Texas Southwestern Medical Center and Children's Medical CenterDallas, Texas, USA; 9 Division of Pediatric Epidemiology and Clinical Research, Department of Pediatrics, University of MinnesotaMinneapolis, Minnesota, USA; 10 Department of Radiation Sciences, Oncology, Umeå UniversityUmea, Sweden; 11 Department of Mathematics and Statistics, University of New MexicoAlbuquerque, New Mexico, USA; 12 Division of Epidemiology and Biostatistics, Department of Internal Medicine, University of New MexicoAlbuquerque, New Mexico, USA; 13 Department of Oncological Sciences, Huntsman Cancer InstituteSalt Lake City, Utah, USA; 14 Department of Pediatrics, University of Utah Health Sciences CenterSalt Lake City, Utah, USA

**Keywords:** Childhood cancer, epidemiology, family history, rhabdomyosarcoma, soft tissue sarcoma

## Abstract

Relatively little is known about the epidemiology and factors underlying susceptibility to childhood rhabdomyosarcoma (RMS). To better characterize genetic susceptibility to childhood RMS, we evaluated the role of family history of cancer using data from the largest case–control study of RMS and the Utah Population Database (UPDB). RMS cases (*n* = 322) were obtained from the Children's Oncology Group (COG). Population-based controls (*n* = 322) were pair-matched to cases on race, sex, and age. Conditional logistic regression was used to evaluate the association between family history of cancer and childhood RMS. The results were validated using the UPDB, from which 130 RMS cases were identified and matched to controls (*n* = 1300) on sex and year of birth. The results were combined to generate summary odds ratios (OR^s^) and 95% confidence intervals (CI). Having a first-degree relative with a cancer history was more common in RMS cases than controls (OR^s^ = 1.39, 95% CI: 0.97–1.98). Notably, this association was stronger among those with embryonal RMS (OR^s^ = 2.44, 95% CI: 1.54–3.86). Moreover, having a first-degree relative who was younger at diagnosis of cancer (<30 years) was associated with a greater risk of RMS (OR^s^ = 2.37, 95% CI: 1.34–4.18). In the largest analysis of its kind, we found that most children diagnosed with RMS did not have a family history of cancer. However, our results indicate an increased risk of RMS (particularly embryonal RMS) in children who have a first-degree relative with cancer, and among those whose relatives were diagnosed with cancer at <30 years of age.

## Introduction

Rhabdomyosarcoma (RMS) is a malignant tumor of skeletal muscle. While RMS is the most common soft tissue sarcoma in children 1, the annual incidence is only 4.6 per million in people younger than 20 years of age 2. In the United States (US), about 350 children and adolescents are diagnosed with RMS per year 3, and half of those cases occur before 10 years of age 2. The two major histologic subtypes of RMS are embryonal (∼70% of cases) and alveolar (∼30% of cases). While embryonal RMS are characterized by loss of heterozygosity/loss of imprinting at loci on chromosome 11p15, ∼80% of alveolar RMS are driven by a specific chromosomal translocation between either of the transcription factors *PAX3* or *PAX7* and *FOXO1* 4–6.

Relatively little is known about the epidemiology and factors underlying susceptibility to childhood RMS. Inherited genetic susceptibility is believed to play an important role in the development of childhood RMS 7. For instance, ∼5% of cases appear to be associated with familial syndromes 8. Specifically, within Li-Fraumeni syndrome (LFS) families that carry germline *TP53* mutations, RMS is one of the most common childhood malignancies 9,10. Additionally, in one report from the fourth trial of the Intergroup Rhabdomyosarcoma Study Group (IRS-IV), the prevalence of neurofibromatosis type 1 was ∼20 times greater in children with RMS compared to the general population (0.5% vs. 0.02–0.03%) 11. In spite of these associations, much work remains in characterizing the role of genetic susceptibility in the etiology of childhood RMS.

Having a family history of cancer has been shown to be associated with childhood cancers including acute lymphoblastic leukemia 12, germ cell tumors 13, Hodgkin lymphoma, and non-Hodgkin lymphoma 14, however, to our knowledge, there have been no systematic population-based studies evaluating the role of family history of cancer in the etiology of childhood RMS. Because family history of cancer is often used to determine the influence of inherited susceptibility in cancer risk, we assessed the association between family history of cancer and RMS in the largest case–control study of childhood RMS to date and the Utah Population Database (UPDB).

## Materials and Methods

### COG discovery cohort

#### Study population

Cases and controls were enrolled from the third trial previously coordinated by the Intergroup Rhabdomyosarcoma Study Group (IRSG), which became part of the Children's Oncology Group (COG) in 2000 and managed treatment protocols for 80–85% of all childhood RMS cases in North America 15. The details regarding the case–control study have been previously described 16–18. Briefly, the cases included those who were 0 years old and up through 20 years of age at the time of their RMS diagnosis from April 1982 to July 1988. Central expert pathology review, coordinated by COG, confirmed all RMS diagnoses, as well as histologic subtype (i.e., embryonal, alveolar, or other). Controls were identified by random-digit dialing during the same period 16–18. Controls were pair-matched to cases on race, sex, and age.

#### Data collection and variables

Data were collected from case and control families by telephone interview using a structured questionnaire, which included items on family cancer history among first- and second-degree relatives. The child's mother and father were asked to participate in the interview, which for case and control families lasted on average 70 and 68 min, respectively. Interviews were conducted in English and Spanish. The Institutional Review Board (IRB) at Baylor College of Medicine approved this study.

### UPDB validation cohort

The UPDB is a dynamic resource located at the University of Utah and consists of computerized statewide vital records, cancer registry information, and administrative claims data for 7.3 million living and deceased individuals, beginning in the early 1900s. Most families living in Utah are represented in the UPDB multigenerational pedigrees. Data from the Utah Cancer Registry (UCR), a Surveillance Epidemiology and End Results (SEER) registry since 1973, are regularly linked to the UPDB. This provides an ongoing and accurate assessment of family history of cancer that does not depend on self-report. We identified RMS cases diagnosed at 0–20 years of age from the UCR from 1966 to 2011. Unaffected population controls were selected randomly from individuals in UPDB and matched 10:1 to RMS cases on sex and birth year. To appropriately match exposure periods, a control had to have follow up at least as long as the date of diagnosis for their respective case. COG cases were neither born nor diagnosed in Utah, and therefore, there was no overlap between cases from the COG and UPDB cohorts. The University of Utah's IRB and Resource for Genetic Epidemiologic Research approved this study.

### Statistical analyses

Descriptive statistics were used to characterize the demographic variables among the case and control groups. To compare the potential prevalence of LFS in RMS cases with previous reports, we determined the proportion of cases that met the revised Chompret criteria 19,20. Specifically, the criteria were met if the case had a first- or second-degree relative diagnosed with (1) at least one tumor classified under the LFS spectrum (e.g., soft tissue sarcoma, osteosarcoma, brain tumor, premenopausal breast cancer, adrenocortical carcinoma, leukemia, lung bronchoalveolar cancer) at <56 years of age, or (2) multiple tumors.

For the COG discovery cohort, conditional logistic regression was used to evaluate cancer history among first- and second-degree relatives and the association with childhood RMS by generating adjusted odds ratios (OR^a^) and 95% confidence intervals (CI). Specifically, cancer history was assessed among first-degree relatives, second-degree relatives, and any relatives (i.e., either a first- or second-degree relative). Stratified analyses were conducted to: (1) evaluate the association of family cancer history and childhood RMS for children who are male and those who are female; (2) children diagnosed under 5 years of age and those diagnosed later (based on sample size and previous assessments) 13; and (3) for children who have relatives diagnosed with a malignancy before the age of 30 years and those with relatives diagnosed when older than 30 years. Because the RMS histologic subtypes are suspected to be heterogeneous in etiology, the association of family cancer history and childhood RMS was also assessed separately for children diagnosed with embryonal RMS; we did not separately assess those with alveolar RMS due to the potential heterogeneity within this group as information on *PAX-FOXO1* fusion status was not available. Finally, the association of family cancer history and childhood RMS was independently evaluated among each cancer type diagnosed in their relatives. All statistical models were adjusted for the matching factors including the child's sex (male or female), age at diagnosis (in years), and race (categorized as White, Black, or other). An association was considered statistically significant if *P* < 0.05.

Analyses were repeated with the UPDB validation cohort using unconditional logistic regression to generate OR^a^ and 95% CIs, adjusting for the matching factors of sex and year of birth. The results from the COG discovery cohort and the UPDB validation cohort were combined using weighted standard errors in meta-analysis, due to differences in study design between the cohorts, to generate summary ORs (OR^s^) and 95% CIs. We tested for heterogeneity across the two studies (i.e., cohorts) using Cochran's *Q*-test 21.

All analyses were conducted using STATA 12.1 (StataCorp LP, College Station, TX) and SAS 9.1.3 (SAS Institute, Cary, NC).

## Results

There were 322 case–control pairs available from the COG discovery cohort and 130 RMS cases and 1300 controls from the UPDB validation cohort for the present analysis (Table1). A higher proportion of case mothers (COG 14.1%, UPDB 13.2%) and fathers (COG 17.1%, UPDB 8.6%) had less than a high school education compared to control mothers (COG 12.2%, UPDB 11.1%) and fathers (COG 11.8%, UPDB 7.0%). Additionally, a higher proportion of cases (COG 32.8%) were from households where the total annual income was less than $20,000 compared to controls (COG 24.3%). The most common histologic subtype of RMS in this population was embryonal (COG 66.7%, UPDB 50.0%) followed by alveolar (COG 20.5%, UPDB 29.2%). The prevalence of potential LFS (when applying the revised Chompret criteria) was similar among RMS cases diagnosed at <3 years of age (COG 13.0%, UPDB 12.0%) and those diagnosed at ≥3 years of age (COG 13.3%, UPDB 14.3%).

**Table 1:** Demographic characteristics among cases and controls.

Characteristic	Children's Oncology Group		Utah Population Database	
	Controls (*n* = 322)	Cases (*n* = 322)	Controls (*n* = 1300)	Cases (*n* = 1300)
Child				
Sex, *n* (%)				
Male	215 (66.8)	215 (66.8)	690 (53.1)	69 (53.1)
Female	107 (33.2)	107 (33.2)	610 (46.9)	61 (46.9)
Race, *n* (%)				
White	291 (90.4)	287 (89.1)	1215 (93.5)	127 (97.7)
Non-white	31 (9.6)	35 (10.9)	85 (6.5)	3 (2.3)
Ethnicity, n (%)				
Non-Hispanic	307 (95.9)	303 (94.7)	1233 (95.9)	122 (93.8)
Hispanic	13 (4.1)	17 (5.3)	53 (4.1)	8 (6.2)
Age at diagnosis/enrollment (years), mean (SD)	7.5 (5.4)	7.6 (5.3)	8.4 (6.2)	8.4 (6.2)
Parents				
Maternal education, *n* (%)				
<High school	39 (12.2)	45 (14.1)	116 (11.1)	15 (13.2)
High school	126 (39.4)	132 (41.4)	342 (32.7)	39 (34.2)
>High school	155 (48.4)	142 (44.5)	589 (56.2)	60 (52.6)
Paternal education, *n* (%)				
<High school	37 (11.8)	54 (17.1)	68 (7.0)	9 (8.6)
High school	111 (35.5)	112 (35.3)	242 (24.9)	24 (23.1)
>High school	165 (52.7)	151 (47.6)	663 (68.1)	71 (68.3)
Annual household income, *n* (%)				
<$20,000	77 (24.3)	104 (32.8)		
$20,000–$39,999	155 (48.9)	131 (41.3)		
≥$40,000	85 (26.8)	82 (25.9)		
Rhabdomyosarcoma				
Histologic subtypes, *n* (%)				
Embryonal		215 (66.7)		65 (50.0)
Alveolar		66 (20.5)		38 (29.2)
NOS		41 (12.8)		27 (20.8)
Potential Li-Fraumeni syndrome^1^				
Diagnosed at <3 years old				
No		65 (86.7)		22 (88.0)
Yes		10 (13.3)		3 (12.0)
Diagnosed at ≥3 years old				
No		215 (87.0)		90 (85.7)
Yes		32 (13.0)		15 (14.3)

NOS, not otherwise specified.

1 Determined using the revised Chompret criteria 19,20.

Most RMS cases did not have a first-degree relative with a history of cancer (COG 92.2%, UPDB 94.5%). While not statistically significant, having any first-degree relative with cancer was positively associated with childhood RMS (Table2) (OR^s^ = 1.39, 95% CI: 0.97–1.98). The direction and magnitude of the association did not differ based on maternal or paternal history of cancer (COG OR^a^ = 1.33, 95% CI: 0.51–3.44 and COG OR^a^ = 1.30, 95% CI: 0.48–3.51, respectively). While there were no statistically significant associations between a family history of specific cancer types and childhood RMS in the COG data (Table S1), there were positive associations with a family history of cancer of the lip or oral cavity (OR^a^ = 2.44, 95% CI: 0.22–27.43), melanoma (OR^a^ = 1.44, 95% CI: 0.24–8.68), breast (OR^a^ = 1.72, 95% CI: 0.62–4.78), and uterus or ovary (OR^a^ = 1.77, 95% CI: 0.41–7.57).

**Table 2:** Family history of cancer in first- and second-degree relatives and risk of childhood rhabdomyosarcoma.

Family cancer history	COG (cases, *n* = 322; controls, *n* = 322)				UPDB (cases, *n* = 130; controls, *n* = 1300)				Combined	
	Cases, *n* (%)	Controls, *n* (%)	OR^1^	95% CI	Cases, *n* (%)	Controls, *n* (%)	OR^1^	95% CI	OR^s^	95% CI
First-degree relative										
Parents										
No	288 (93.8)	293 (95.4)	1.0	Reference	95 (80.5)	932 (84.9)	1.0	Reference	1.0	Reference
Yes	19 (6.2)	14 (4.6)	1.42	0.71–2.85	23 (19.5)	166 (15.1)	1.34	0.85–2.11	1.36	0.93–2.0
Siblings										
No	238 (99.6)	238 (99.6)	1.0	Reference	98 (97.0)	857 (96.8)	1.0	Reference	1.0	Reference
Yes	1 (0.4)	1 (0.4)	NE^2^	NE^2^	3 (3.0)	28 (3.2)	1.42	0.54–3.72	NE^2^	NE^2^
Any										
No	248 (92.2)	262 (94.6)	1.0	Reference	96 (80.0)	983 (84.6)	1.0	Reference	1.0	Reference
Yes	21 (7.8)	15 (5.4)	1.46	0.72–2.97	24 (20.0)	179 (15.4)	1.36	0.90–2.06	1.39	0.97–1.98
Second-degree relative^3^										
No	137 (47.9)	132 (46.2)	1.0	Reference	40 (38.5)	388 (41.6)	1.0	Reference	1.0	Reference
Yes	149 (52.1)	154 (53.8)	0.92	0.66–1.29	64 (61.5)	544 (58.4)	1.13	0.90–1.14	1.11	0.99–1.24
First- or second-degree relative										
No	98 (39.5)	97 (39.1)	1.0	Reference	44 (36.7)	560 (48.2)	1.0	Reference	1.0	Reference
Yes	150 (60.5)	151 (60.9)	0.98	0.68–1.42	76 (63.3)	602 (51.8)	1.18	0.97–1.43	1.13	0.96–1.35

COG, Children's Oncology Group; UPDB, Utah Population Database; OR, odds ratio; NE, not estimated.

1 COG, adjusted for sex, age, and race; UPDB, adjusted for sex and year of birth.

2 COG, not estimated due to small cells; Combined, not estimated because unable to generate estimates for the COG cohort.

3 COG, includes grandparents, aunts, and uncles; UPDB, also includes nieces and nephews.

Stratified analyses (Table3) revealed that if the first-degree relative was <30 years of age when diagnosed with cancer, the association between family history of cancer and childhood RMS was stronger than if the first-degree relative was ≥30 years of age at diagnosis, (COG OR^a^ = 1.69, 95% CI: 0.76–3.78 vs. OR^a^ = 1.32, 95% CI: 0.58–3.01; UPDB OR^a^ = 3.33, 95% CI: 1.48–7.46 vs. OR^a^ = 1.14, 95% CI: 0.71–1.83). Additionally, when combining the COG and UPDB results, having a first-degree relative diagnosed at <30 years of age was significantly associated with RMS risk (OR^s^ = 2.37, 95% CI: 1.34–4.18). In order to determine if this finding was driven in part to LFS, we restricted our analysis to those who did not meet the Chompret criteria (COG OR^a^ = 2.02, 95% CI: 0.67–6.09).

**Table 3:** Family history of cancer in first- or second-degree relatives and risk of childhood rhabdomyosarcoma: stratified results.

Family cancer history	COG (cases, *n* = 322; controls, *n* = 322)				UPDB (cases, *n* = 130; controls, *n* = 1300)				Combined	
	Cases, *n* (%)	Controls, *n* (%)	OR^1^	95% CI	Cases, *n* (%)	Controls, *n* (%)	OR^1^	95% CI	OR^s^	95% CI
First-degree relative										
Child's sex										
Male	15 (9.2)	9 (5.5)	1.55	0.67–3.60	10 (8.3)	103 (8.9)	0.83	0.40–1.56	1.06	0.63–1.80
Female	5 (7.1)	4 (5.7)	1.32	0.34–5.15	14 (11.7)	76 (6.5)	2.02	1.16–3.39	1.91	1.16–3.14
Child's age at diagnosis										
<5 years	5 (7.0)	3 (4.2)	1.37	0.29–6.35	7 (5.8)	48 (4.1)	1.68	0.76–3.40	1.62	0.82–3.17
≥5 years	13 (9.4)	10 (7.2)	1.29	0.56–2.94	17 (14.2)	131 (11.3)	1.23	0.73–1.99	1.25	0.81–1.91
Relatives’ youngest age at diagnosis										
<30 years	6 (2.9)	2 (1.0)	1.69	0.76–3.78	5 (4.2)	20 (1.7)	3.33	1.48–7.46	2.37	1.34–4.18
≥30 years	13 (5.8)	10 (4.5)	1.32	0.58–3.01	19 (16.7)	166 (14.3)	1.14	0.71–1.83	1.18	0.78–1.78
Second-degree relative^2^										
Child's sex										
Male	108 (57.5)	105 (55.9)	1.05	0.69–1.61	33 (31.7)	293 (31.4)	1.27	0.93–1.72	1.19	0.93–1.53
Female	41 (41.8)	49 (50.0)	0.77	0.44–1.33	31 (29.8)	251 (26.9)	1.01	0.74–1.37	0.95	0.72–1.24
Child's age at diagnosis										
<5 years	48 (44.9)	49 (45.8)	1.02	0.58–1.78	12 (11.5)	156 (16.7)	0.68	0.41–1.08	0.81	0.56–1.17
≥5 years	85 (57.8)	90 (61.9)	0.85	0.52–1.37	52 (50.0)	388 (41.6)	1.33	1.04–1.70	1.21	0.98–1.51
Relatives’ youngest age at diagnosis										
<30 years	9 (11.5)	5 (6.4)	1.38	0.78–2.42	3 (2.9)	29 (3.1)	1.14	0.40–3.24	1.32	0.80–2.17
≥30 years	115 (47.3)	122 (50.2)	0.89	0.62–1.27	63 (60.6)	529 (56.8)	1.13	0.91–1.41	1.06	0.88–1.28
First- or second-degree relative										
Child's sex										
Male	111 (66.1)	104 (61.9)	1.21	0.76–1.93	37 (30.8)	323 (27.8)	1.18	0.88–1.54	1.19	0.94–1.51
Female	39 (48.8)	47 (58.8)	0.71	0.37–1.36	26 (21.7)	290 (25.0)	1.20	0.91–1.56	1.11	0.87–1.43
Child's age at diagnosis										
<5 years	46 (54.8)	46 (54.8)	1.06	0.57–1.97	18 (15.0)	169 (14.5)	0.86	0.56–1.27	0.92	0.65–1.29
≥5 years	91 (65.9)	92 (66.7)	1.00	0.60–1.67	58 (48.3)	433 (37.3)	1.32	1.06–1.64	1.27	1.04–1.55
Relatives’ youngest age at diagnosis										
<30 years	10 (18.9)	5 (9.4)	1.43	0.78–2.61	7 (5.8)	28 (2.4)	2.07	1.10–3.86	1.71	1.11–2.64
≥30 years	110 (55.0)	115 (57.5)	0.95	0.63–1.42	72 (60.0)	586 (50.4)	1.14	0.93–1.39	1.10	0.92–1.32

COG, Children's Oncology Group; UPDB, Utah Population Database; OR, odds ratio.

1 COG, adjusted for sex, age, and race; UPDB, adjusted for sex and year of birth.

2 COG, Includes grandparents, aunts, and uncles; UPDB, also includes nieces and nephews.

When assessing embryonal RMS and the influence of family history of cancer on disease occurrence (Table4), there was a positive association between having a first-degree relative with cancer and embryonal RMS (COG OR^a^ = 1.58, 95% CI: 0.61–4.10), with a strong and statistically significant association detected in the UPDB cohort (OR^a^ = 2.78, 95% CI: 1.22–3.50). When combining the COG and UPDB results, having a first-degree relative with a history of cancer was significantly associated with embryonal RMS (OR^s^ = 2.44, 95% CI: 1.54–3.86). There was no association between having a second-degree relative with cancer and embryonal RMS in the COG cohort, however, a positive nonsignificant association was detected in the UPDB cohort (OR^a^ = 1.21, 95% CI: 0.89–1.63).

**Table 4:** Associations of family history of cancer and embryonal rhabdomyosarcoma.

Family cancer history	COG, *n* = 215				UPDB, *n* = 65				Combined	
	Cases, *n* (%)	Controls, *n* (%)	OR^1^	95% CI	Cases, *n* (%)	Controls, *n* (%)	OR^1^	95% CI	OR^1^	95% CI
First-degree relative	12 (8.2)	7 (4.8)	1.58	0.61–4.10	14 (11.7)	87 (7.5)	2.78	1.22–3.50	2.44	1.54–3.86
Second-degree relative^2^	103 (53.9)	105 (55.0)	0.97	0.64–1.46	33 (31.7)	279 (29.2)	1.21	0.89–1.63	1.12	0.88–1.43
Any first- or second-degree relative	100 (61.4)	100 (61.4)	1.04	0.66–1.64	76 (63.3)	602 (51.8)	1.38	1.06–1.79	1.29	1.03–1.61

COG, Children's Oncology Group; UPDB, Utah Population Database; OR, odds ratio.

1 COG, adjusted for sex, age, and race; UPDB, adjusted for sex and year of birth.

2 COG, Includes grandparents, aunts, and uncles; UPDB, also includes nieces and nephews.

There was no heterogeneity detected between the COG and UPDB cohorts when combining results to generate OR^s^ estimates (*P* for heterogeneity >0.100).

## Discussion

In the largest analysis of its kind to date, we found that most RMS cases did not have a first-degree relative with a history of cancer. However, three patterns emerged: (1) having any first-degree relative with a history of cancer was more common in RMS cases than controls; (2) having a first-degree relative who was younger (<30 years of age) when diagnosed with cancer was more strongly associated with childhood RMS than having a first-degree relative who was older at diagnosis (≥30 years of age); and (3) having a first-degree relative with cancer was strongly associated with embryonal RMS.

While there have been no previous population-based studies of family history of cancer and childhood RMS, our results are consistent with previously reported associations between family history of cancer and other childhood cancers. For instance, in a case–control study conducted in Canada, the authors reported a positive but nonsignificant association between a family history of cancer among first-degree relatives and childhood acute lymphoblastic leukemia (OR = 1.2, 95% CI: 0.6–2.3) 12. Data from the French national population-based ESCALE study indicated that a family history of cancer was associated with an increased risk of Hodgkin lymphoma (OR = 1.5, 95% CI: 1.0–2.2) and non-Hodgkin lymphoma (OR = 1.8, 95% CI: 1.3–2.5) 14. The magnitude of these associations is similar to our findings. As in our study, the ESCALE study reported associations were stronger when the relative was first-degree (e.g., Hodgkin lymphoma OR = 2.2, 95% CI: 0.9–5.1) versus second degree (OR = 1.4, 95% CI: 1.0–2.1). Additionally, the ORs were higher when relatives were diagnosed earlier in life (<46 years of age), which was also the case in our population. This is further supported by a report from the COG where the association between family history of cancer and germ cell tumors in male children was stronger when the relative was <40 years of age at diagnosis (OR = 2.6, 95% CI: 1.0–6.44) compared to when the relative was 40–49 years of age at diagnosis (OR = 1.2, 95% CI: 0.5–3.4) or ≥50 years of age at diagnosis (OR = 1.3, 95% CI: 0.60–2.73) 13.

Case reports and case series of childhood RMS have indicated that a family history of cancer or of a cancer-predisposing syndrome is an important factor in disease risk. Li and Fraumeni reported that among 648 childhood RMS cases, four were from families in which siblings or cousins had a childhood sarcoma 22. These families also had histories of breast cancer and other neoplasms. While not statistically significant, in our population, a family history of breast cancer was positively associated with childhood RMS (OR^a^ = 1.72, 95% CI: 0.62–4.78). Among children who were <3 years of age at diagnosis, 13.3% and 12.0% had a family history of cancer consistent with that of LFS in the COG and UPDB cohorts, respectively. This supports previous reports that estimate 10–15% of younger children (i.e., <3 years of age) diagnosed with RMS may have LFS 23. Additionally, in our data ∼13–14% children ≥3 years of age at diagnosis also met the Chompret criteria for potential LFS. This is in contrast to a previous report which suggested that LFS may not be as common among those older than 3 years of age at diagnosis 24. Furthermore, these estimates were confirmed in the UPDB cohort.

Our results further indicate that the RMS risk among children with first-degree relatives that were younger at cancer diagnosis (<30 years of age) was not driven by LFS. This could indicate that other cancer susceptibility genes that are yet to be identified may underlie RMS.

As indicated, in our study, family history of cancer was more strongly associated with embryonal RMS than when assessing all RMS cases together. This is notable as embryonal RMS is characterized by a younger age at onset compared to alveolar RMS 25, and there is some evidence that embryonal RMS is more common than alveolar RMS in families with *TP53* mutations 26–28. Interestingly, anaplastic RMS also appears to be associated with germline *TP53* mutations 29. Unfortunately, anaplastic histology was not annotated in IRS-III or the UPDB. Lastly, in a hospital-based survey, investigators observed that relatives of sarcoma patients were more likely to have an excess of cancer when the sarcoma histologic type was embryonal RMS 30,31. This may point to a stronger role of family history of cancer in the development of embryonal RMS when compared with alveolar RMS; however, this must be further investigated.

Our results should be considered in light of certain limitations. First, family history of disease was obtained by self-report for the COG cohort. Self-report of family history of cancer is relatively accurate in case–control studies; however, reliability appears to be higher for reports for first-degree relatives compared to more distant relatives 32–35. Although we evaluated associations between both first- (parents and siblings) and second-degree (grandparents and aunts/uncles) relatives, as parents provided family history information about their first-degree relatives (i.e., the child's grandparents and aunts/uncles), we might expect the information about a child's second-degree relative to be more accurate than in comparable studies of adult cancers. Parents of cases might also be expected to give a more thorough history than control parents, although this is not supported by three previous validation studies 33,36,37. Lastly, our results were validated using the UPDB, which is a population-based resource that relies on record linkages between birth certificates, the UCR, and medical records to follow cancer diagnoses through family pedigrees. Associations found in the UPDB cohort were consistent, and sometimes stronger when compared with the COG cohort (i.e., the OR for having a first-degree relative with cancer among those with embryonal RMS was 76% stronger in the UPDB compared to the COG cohort).

Another limitation is that while this is the largest case–control study of childhood RMS to date, we were restricted to evaluating only first- and second-degree relatives. Additionally, due to small numbers, it was not possible to assess disease risk associated with increasing number of relatives with a previous cancer diagnosis. For instance, in the COG population, less than 1% of subjects had two first-degree relatives with a history of cancer.

Recent findings confirm that ∼20% of alveolar RMS tumors do not exhibit a *PAX-FOXO1* rearrangement 38, and that “fusion negative” alveolar RMS cases have clinical outcomes similar to those with embryonal RMS 6,39,40. In fact, the biology of fusion negative alveolar RMS tumors may be closer to embryonal RMS tumors, suggesting these two phenotypes could be considered together in epidemiologic assessments. Unfortunately, *PAX-FOX01* fusions were not assessed when the COG cases were diagnosed in the 1980s; therefore it is not possible to evaluate the influence of family cancer history on RMS based on fusion status. However, it is not clear if risk factors for embryonal RMS and fusion negative alveolar RMS overlap. Furthermore, several previous epidemiologic assessments have evaluated embryonal RMS as a distinct phenotype 9,16,25,41.

This study has several major strengths. First, this is the largest case–control study to evaluate the influence of family history of cancer on childhood RMS, with 322 childhood RMS cases from the COG cohort. Second, this study is unique in that it provides a population-based estimate of potential LFS among those with RMS. Lastly, we validated our findings (COG) in a second independent cohort (UPDB). While this is common for large-scale genetic studies, it is not typically practiced in classical epidemiologic assessments.

While only a minority of children with RMS had a family history of cancer, this study adds to the body of evidence that inherited genetic susceptibility may be a factor in the development of childhood RMS. This is reflected in a modest increase (i.e., 39%) in familial cancer incidence and earlier onset of these malignancies. Much work remains in characterizing germline genetic susceptibility to childhood RMS. Unlike many other childhood cancers, there have been few germline candidate gene studies of RMS and no genome-wide association studies to date. As little is known about the epidemiology of childhood RMS, it will be important to further examine the genetic underpinnings of these complex phenotypes in future studies.

## Supporting Information

**Table S1.** Family history of cancer types and risk of childhood RMS.

## References

[b1] Li J, Thompson TD, Miller JW, Pollack LA, Stewart SL (2008). Cancer incidence among children and adolescents in the United States, 2001–2003. Pediatrics.

[b2] Gurney JG, Young JL, Roffers SD, Smith MA, Bunin GR (1999). Soft tissue sarcomas. Cancer incidence and survival among children and adolescents: United States SEER Program 1975–1995.

[b3] Hawkins DS, Spunt SL, Skapek SX (2013). Children's Oncology Group's 2013 blueprint for research: soft tissue sarcomas. Pediatr. Blood Cancer.

[b4] Newton WA, Gehan EA, Webber BL, Marsden HB, van Unnik AJ, Hamoudi AB (1995). Classification of rhabdomyosarcomas and related sarcomas. Pathologic aspects and proposal for a new classification—an Intergroup Rhabdomyosarcoma Study. Cancer.

[b5] Barr FG (2001). Gene fusions involving PAX and FOX family members in alveolar rhabdomyosarcoma. Oncogene.

[b6] Williamson D, Missiaglia E, de Reyniès A, Pierron G, Thuille B, Palenzuela G (2010). Fusion gene-negative alveolar rhabdomyosarcoma is clinically and molecularly indistinguishable from embryonal rhabdomyosarcoma. J. Clin. Oncol.

[b7] Ognjanovic S, Carozza SE, Chow EJ, Fox EE, Horel S, McLaughlin CC (2010). Birth characteristics and the risk of childhood rhabdomyosarcoma based on histological subtype. Br. J. Cancer.

[b8] Hemminki K, Li X (2001). A population-based study of familial soft tissue tumors. J. Clin. Epidemiol.

[b9] Ognjanovic S, Olivier M, Bergemann TL, Hainaut P (2012). Sarcomas in TP53 germline mutation carriers: a review of the IARC TP53 database. Cancer.

[b10] Malkin D, Li FP, Strong LC, Fraumeni JF, Nelson CE, Kim DH (1990). Germ line p53 mutations in a familial syndrome of breast cancer, sarcomas, and other neoplasms. Science.

[b11] Sung L, Anderson JR, Arndt C, Raney RB, Meyer WH, Pappo AS (2004). Neurofibromatosis in children with Rhabdomyosarcoma: a report from the Intergroup Rhabdomyosarcoma study IV. J. Pediatr.

[b12] Infante-Rivard C, Guiguet M (2004). Family history of hematopoietic and other cancers in children with acute lymphoblastic leukemia. Cancer Detect. Prev.

[b13] Poynter JN, Radzom AH, Spector LG, Puumala S, Robison LL, Chen Z (2010). Family history of cancer and malignant germ cell tumors in children: a report from the Children's Oncology Group. Cancer Causes Control.

[b14] Rudant J, Menegaux F, Leverger G, Baruchel A, Nelken B, Bertrand Y (2007). Family history of cancer in children with acute leukemia, Hodgkin's lymphoma or non-Hodgkin's lymphoma: the ESCALE study (SFCE). Int. J. Cancer.

[b15] Grufferman S, Delzell E, Delong ER (1984). An approach to conducting epidemiologic research within cooperative clinical trials groups. J. Clin. Oncol.

[b16] Grufferman S, Ruymann F, Ognjanovic S, Erhardt EB, Maurer HM (2009). Prenatal X-ray exposure and rhabdomyosarcoma in children: a report from the Children's Oncology Group. Cancer Epidemiol. Biomarkers Prev.

[b17] Grufferman S, Schwartz AG, Ruymann FB, Maurer HM (1993). Parents’ use of cocaine and marijuana and increased risk of rhabdomyosarcoma in their children. Cancer Causes Control.

[b18] Yang P, Grufferman S, Khoury MJ, Schwartz AG, Kowalski J, Ruymann FB (1995). Association of childhood rhabdomyosarcoma with neurofibromatosis type I and birth defects. Genet. Epidemiol.

[b19] Chompret A, Abel A, Stoppa-Lyonnet D, Brugiéres L, Pagés S, Feunteun J (2001). Sensitivity and predictive value of criteria for p53 germline mutation screening. J. Med. Genet.

[b20] Tinat J, Bougeard G, Baert-Desurmont S, Vasseur S, Martin C, Bouvignies E (2009). 2009 version of the Chompret criteria for Li Fraumeni syndrome. J. Clin. Oncol.

[b21] Cochran W (1954). The combination of estimates from different experiments. Biometrics.

[b22] Li FP, Fraumeni JF (1969). Soft-tissue sarcomas, breast cancer, and other neoplasms. A familial syndrome?. Ann. Intern. Med.

[b23] Plon S, Malkin D, Pizzo P, Poplack D (2010). Childhood cancer & heredity. Principles and practice of pediatric oncology.

[b24] Diller L, Sexsmith E, Gottlieb A, Li FP, Malkin D (1995). Germline p53 mutations are frequently detected in young children with rhabdomyosarcoma. J. Clin. Invest.

[b25] Ognjanovic S, Linabery AM, Charbonneau B, Ross JA (2009). Trends in childhood rhabdomyosarcoma incidence and survival in the United States, 1975–2005. Cancer.

[b26] Ariffin H, Martel-Planche G, Daud SS, Ibrahim K, Hainaut P (2008). Li-Fraumeni syndrome in a Malaysian kindred. Cancer Genet. Cytogenet.

[b27] Ognjanovic S, Martel G, Manivel C, Olivier M, Langer E, Hainaut P (2012). Low prevalence of TP53 mutations and MDM2 amplifications in pediatric rhabdomyosarcoma. Sarcoma.

[b28] Wozniak A, Fryer A, Grimer R, Mc Dowell H (2011). Multiple malignancies in a child with de novo TP53 mutation. Pediatr. Hematol. Oncol.

[b29] Hettmer S, Archer NM, Somers GR, Novokmet A, Wagers AJ, Diller L (2014). Anaplastic rhabdomyosarcoma in TP53 germline mutation carriers. Cancer.

[b30] Lustbader ED, Williams WR, Bondy ML, Strom S, Strong LC (1992). Segregation analysis of cancer in families of childhood soft-tissue-sarcoma patients. Am. J. Hum. Genet.

[b31] Strong LC, Stine M, Norsted TL (1987). Cancer in survivors of childhood soft tissue sarcoma and their relatives. J. Natl. Cancer Inst.

[b32] Airewele G, Adatto P, Cunningham J, Mastromarino C, Spencer C, Sharp M (1998). Family history of cancer in patients with glioma: a validation study of accuracy. J. Natl. Cancer Inst.

[b33] Kerber RA, Slattery ML (1997). Comparison of self-reported and database-linked family history of cancer data in a case-control study. Am. J. Epidemiol.

[b34] Love RR, Evans AM, Josten DM (1985). The accuracy of patient reports of a family history of cancer. J. Chronic Dis.

[b35] Murff HJ, Spigel DR, Syngal S (2004). Does this patient have a family history of cancer? An evidence-based analysis of the accuracy of family cancer history. JAMA.

[b36] Mitchell RJ, Brewster D, Campbell H, Porteous ME, Wyllie AH, Bird CC (2004). Accuracy of reporting of family history of colorectal cancer. Gut.

[b37] Infante-Rivard C, Roncarolo F, Doucette K (2012). Reliability of cancer family history reported by parents in a case-control study of childhood leukemia. Cancer Causes Control.

[b38] Rudzinski ER, Teot LA, Anderson JR, Moore J, Bridge JA, Barr FG (2013). Dense pattern of embryonal rhabdomyosarcoma, a lesion easily confused with alveolar rhabdomyosarcoma: a report from the Soft Tissue Sarcoma Committee of the Children's Oncology Group. Am. J. Clin. Pathol.

[b39] Skapek SX, Anderson J, Barr FG, Bridge JA, Gastier-Foster JM, Parham DM (2013). PAX-FOXO1 fusion status drives unfavorable outcome for children with rhabdomyosarcoma: a Children's Oncology Group report. Pediatr. Blood Cancer.

[b40] Missiaglia E, Williamson D, Chisholm J, Wirapati P, Pierron G, Petel F (2012). PAX3/FOXO1 fusion gene status is the key prognostic molecular marker in rhabdomyosarcoma and significantly improves current risk stratification. J. Clin. Oncol.

[b41] Ognjanovic S, Puumala S, Spector LG, Smith FO, Robison LL, Olshan AF (2009). Maternal health conditions during pregnancy and acute leukemia in children with Down syndrome: a Children's Oncology Group study. Pediatr. Blood Cancer.

