# Supplementary material for: Family history of cancer and childhood rhabdomyosarcoma: a report from the Children's Oncology Group and the Utah Population Database
Source: Cancer Med. 2015 Mar 23;4(5):781–90. doi: 10.1002/cam4.448 (PMC4430270; doi:10.1002/cam4.448)
Supplement: Supplementary file 1 — Table S1. Family history of cancer types and risk of childhood RMS. [file cam40004-0781-sd1.docx]

**Supplemental Table. Family history of cancer types and risk of childhood RMS**

| **Family history of cancer type** | **ICD9 Code** | **Total, n (%)** | |  | **Controls, n (%)** | |  | **Cases, n (%)** | | **OR^a^** | **95% CI** |
| --- | --- | --- | --- | --- | --- | --- | --- | --- | --- | --- | --- |
|  |  | **No** | **Yes** |  | **No** | **Yes** |  | **No** | **Yes** |  |  |
| Lip, oral cavity | 140-149 | 630 (95.4) | 10 (1.6) |  | 318 (99.1) | 3 (0.9) |  | 312 (97.8) | 7 (2.2) | 2.44 | 0.22-27.43 |
| Esophagus, stomach | 150-152 | 613 (95.8) | 27 (4.2) |  | 308 (96.0) | 13 (4.0) |  | 305 (95.6) | 14 (4.4) | 1.09 | 0.22-5.50 |
| Colon, rectum | 153-154 | 599 (93.6) | 41 (6.4) |  | 300 (93.5) | 21 (6.5) |  | 299 (93.7) | 20 (6.3) | 0.91 | 0.30-2.75 |
| Liver, gallbladder, pancreas | 155-157 | 618 (96.6) | 22 (3.4) |  | 309 (96.3) | 12 (3.7) |  | 309 (96.9) | 10 (3.1) | 0.75 | 0.10-5.56 |
| Lung | 162 | 590 (92.2) | 50 (7.8) |  | 297 (92.5) | 24 (7.5) |  | 293 (91.9) | 26 (8.2) | 1.19 | 0.30-4.67 |
| Bone, articular cartilage | 170 | 627 (98.0) | 13 (2.0) |  | 314 (97.8) | 7 (2.2) |  | 313 (98.1) | 6 (1.9) | 1.13 | 0.22-5.67 |
| Connective, other soft tissue | 171 | 636 (99.4) | 4 (0.6) |  | 320 (99.7) | 1 (0.3) |  | 316 (99.1) | 3 (0.9) | -- | -- |
| Melanoma | 172 | 623 (97.3) | 17 (2.7) |  | 313 (97.5) | 8 (2.5) |  | 310 (97.2) | 9 (2.8) | 1.44 | 0.24-8.68 |
| Non-melanoma skin neoplasm | 173 | 594 (92.8) | 46 (7.2) |  | 294 (91.6) | 27 (8.4) |  | 300 (94.0) | 19 (6.0) | 0.55 | 0.20-1.49 |
| Breast | 174 | 576 (90.0) | 64 (10.0) |  | 291 (90.7) | 30 (9.3) |  | 285 (89.3) | 34 (10.7) | 1.72 | 0.62-4.78 |
| Uterus, ovary | 179-184 | 592 (92.5) | 48 (7.5) |  | 298 (92.8) | 23 (7.2) |  | 294 (92.2) | 25 (7.8) | 1.77 | 0.41-7.57 |
| Prostate | 185 | 616 (96.3) | 24 (3.8) |  | 309 (96.3) | 12 (3.7) |  | 307 (96.2) | 12 (3.8) | 0.44 | 0.04-4.37 |
| Bladder | 188-189 | 628 (98.1) | 12 (1.9) |  | 315 (98.1) | 6 (1.9) |  | 313 (98.1) | 6 (1.9) | 0.75 | 0.10-5.57 |
| Brain | 191 | 615 (96.1) | 25 (3.9) |  | 311 (96.9) | 10 (3.1) |  | 304 (95.3) | 15 (4.7) | 0.90 | 0.18-4.56 |
| Lymphatic & hematopoietic tissue | 200-208 | 605 (94.5) | 35 (5.5) |  | 299 (93.2) | 22 (6.8) |  | 306 (95.9) | 13 (4.1) | 0.79 | 0.23-2.69 |
| Other |  | 590 (92.2) | 50 (7.8) |  | 297 (92.5) | 24 (7.5) |  | 293 (91.9) | 26 (8.1) | 1.37 | 0.43-4.39 |
| ^a^ Adjusted for the matching factors of sex, age, and race. | | | | | | | | | | | |
| OR=odds ratio | | | | | | | | | | |  |
